# Selenium supplementation in radiotherapy patients: do we need to measure selenium levels in serum or blood regularly prior radiotherapy?

**DOI:** 10.1186/s13014-014-0289-0

**Published:** 2014-12-16

**Authors:** Ralph Muecke, Oliver Micke, Lutz Schomburg, Klaus Kisters, Jens Buentzel, Jutta Huebner, Jan Kriz

**Affiliations:** Department of Radiotherapy, Lippe Hospital Lemgo, Rintelner Str. 85, D-32657 Lemgo, Germany; Department of Radiotherapy and Radiation Oncology, Ruhr University Bochum, Bochum, Germany; Department of Radiotherapy and Radiation Oncology, Franziskus Hospital, Bielefeld, Germany; Institute for Experimental Endocrinology, Charité Berlin, Germany; Department of Internal Medicine, St. Anna Hospital, Herne, Germany; Department of Otolaryngology, Südharz Hospital, Nordhausen, Germany; Working Group Integrative Oncology, German Cancer Society, Berlin, Germany; Department of Radiotherapy and Radiation Oncology, Münster University Hospital, Münster, Germany

**Keywords:** Selenium, Supplementation, Clinical studies, Selenoproteins, Radiotherapy

## Abstract

Considering the review by Puspitasari and colleagues, an additional discussion of the endpoints of the Se supplementation studies described would be helpful. In our view, selenium can safely be given to selenium-deficient cancer patients prior to and during radiotherapy. Therefore, in order to help the radiation oncologist in decision making, we strongly advocate to determine the selenium status prior to and during a potential adjuvant selenium supplementation, e.g. when trying to ease the side-effects of radiation treatment or in the aftercare situation when the selenium status may become insufficient.

We have read with great interest the review of Puspitasari et al. and would like to thank the authors for this interesting summary which shows the possible benefits of selenium supplementation in radiotherapy patients reducing side effects and thus improving quality of life without compromising the effectiveness of radiotherapy [1].

In the introduction, the authors point to the necessity of a guideline helping physicians and patients with their decisions concerning selenium supplementation. They correctly point to the lack of comprehensive data. Yet, if one enlarges the range of their systematic search, there are additional data which to our opinion could form the basis of practice rules concerning selenium in cancer care. In fact, there is a wealth of preclinical and clinical data pointing to the dose-dependency of health effects from selenium supplementation and the high mortality risk of selenium-deficient cancer patients [2].

According to recently published studies it seems most important for cancer patients to achieve and maintain a certain range of Se in the serum. Nutritional Se intake, plasma Se concentration and glutathione peroxidase (GPx) activity display a positive correlation up to a certain threshold plasma Se concentration (70–100 μg/L), beyond which the GPx activity plateaus [3]. The limit at which selenoprotein P (SePP) concentrations may no longer increase with higher Se intake has been determined at levels of around 90–125 μg Se/l plasma [4-6]. Consequently, the optimal Se range in serum likely resides between 100 and 130 μg/l [7,8]. This is in accordance with epidemiological and clinical data which underline that cancer and mortality risks inversely correlate to Se concentrations at suboptimal levels <100 μg Se/l plasma as compared to higher Se status [9].

Considering the review by Puspitasari and colleagues, an additional discussion of the endpoints of the Se supplementation studies described would be helpful. In order to determine effects from selenium basic and follow-up levels need to be determined. Unfortunately, some well-known and frequently cited allegedly negative studies did not obey to these simple rules and it is thus difficult to interpret their findings [10,11].

Therefore, in order to help the radiation oncologist in decision making, we strongly advocate to determine the selenium status prior to and during a potential adjuvant selenium supplementation, e.g. when trying to ease the side-effects of radiation treatment or in the aftercare situation when the selenium status may become insufficient. The potential benefits for the cancer patient under radiotherapy are well-documented and undisputed [1,2]. In our view, selenium can safely be given to selenium-deficient cancer patients prior to and during radiotherapy. Considering the known uncertainties around the appropriate Se dose and Se compound we would propose the inorganic form sodium selenite without unspecific incorporation into non-selenoenzymes and daily doses higher than in the above mentioned gynecological study of 500 μg since the patients with Se supplementation did not achieve Se levels in the serum higher than 75 μg/l [12]. Finally we want to point out that selenium is not a medication per se but rather an adjuvant treatment option supporting the biosynthesis of 25 human genes encoding the functionally active selenoproteins needed for limiting the side effect of radiation therapy and supporting a fast recovery.
